# Effects and Mechanisms of *Dendrobium officinalis* Six Nostrum for Treatment of Hyperuricemia with Hyperlipidemia

**DOI:** 10.1155/2020/2914019

**Published:** 2020-03-30

**Authors:** Lin-Feng Guo, Xue Chen, Shan-Shan Lei, Bo Li, Ning-Yu Zhang, Hong-Zhang Ge, Ke Yang, Gui-Yuan Lv, Su-Hong Chen

**Affiliations:** ^1^Collaborative Innovation Center of Yangtze River Delta Region Green Pharmaceuticals, Zhejiang University of Technology, Hangzhou, Zhejiang 310014, China; ^2^College of Pharmaceutical Science, Zhejiang Chinese Medical University, Hangzhou, Zhejiang 310014, China

## Abstract

*Objectives*. Hyperuricemia (HUA) is a disease caused by increased production of uric acid (UA) or reduced excretion of UA in the body. Results of an epidemiological survey show that 60% of patients with HUA have hyperlipidemia (HPA). *Dendrobium officinalis* (DOF) six nostrum (DOS) is based on the theory of traditional Chinese medicine for the transformation of the traditional Chinese nostrum Si Miao Wan. In this article, we aim to discuss the efficacy and mechanism of DOS in reducing UA and regulating lipid metabolism. The rat model of HUA with HPA was induced by potassium oxonate (PO) combined with high-fat sorghum feed. We monitored the serum UA and blood lipids. Liver xanthine oxidase (XOD), adenosine deaminase (ADA), lipoprotein lipase (LPL), and fatty acid-binding protein (FABP1) activities were measured by enzyme-linked immunosorbent assay (ELISA) after the last administration of DOS. We performed a histopathological examination of rat kidney and intestine. Immunohistochemistry (IHC) was used to detect the expression of renal inflammatory proteins NLRP3 / Caspase-1 and intestinal inflammatory proteins TLR4 / NLRP3. We used western blot for measurement of liver hypoxanthine-guanine phosphoribosyl transferase (HPRT1) protein expression and renal PDZ domain protein kidney 1 (PDZK1) protein expression. DOS administration significantly reduced serum UA, total cholesterol (TC), and low-density lipoprotein cholesterol (LDL-c) level, and improved liver steatosis in the model rat. At the same time, DOS treatment effectively inhibited liver XOD and ADA, increased the level of liver HPRT1, and reduced the production of UA. Additional studies had shown that DOS can restore normal UA excretion function in the intestine and kidney and regulated liver lipids metabolism. IHC and histopathological sections showed that DOS reduced the level of kidney, intestinal inflammatory body (NLRP3, Caspase-1, and TLR4), improved inflammation of the kidney and intestinal tract in rats. DOS is a promising drug that can effectively reduce serum UA and lipid level in the model rat. The mechanism of action may be related to inhibition of UA production, promotion of UA excretion, regulation of lipids metabolism, and anti-inflammatory response.

## 1. Introduction

Hyperuricemia (HUA) is often caused by hereditary and (or) acquired causes of decreased uric acid (UA) excretion or increased UA production. Both of these scenarios cause UA to accumulate in the body, resulting in a continuous increase in blood UA [1]. In recent years, the incidence of global HUA has been increasing year by year. HUA has become the fourth major risk factor after hypertension, hyperlipidemia (HPA), and hyperglycemia [2]. HUA patients with HPA cases are typical in the clinic setting [3]. The results of an epidemiological survey showed that about 60% of patients with HUA had abnormal lipid metabolism and later developed HUA with HPA [4]. Elevated serum uric acid (SUA) aggravates lipid metabolism disorders and increases the risk of high low-density lipoprotein cholesterol (LDL-c) [5], while abnormal lipid metabolism can also aggravate SUA level [6]. It can be hypothesized that abnormal UA metabolism and abnormal serum lipid metabolism interact in the body to form a vicious circle, which leads to aggravation in patients with HUA and HPA.

At present, multiple drugs are often used to treat HUA with HPA [7, 8]. The combination of an antihyperuricemic (e.g., febuxostat and allopurinol) and a hypolipidemic drug (e.g., statins) has a risk of overlapping adverse drug reactions while reducing SUA and lipid level [9, 10], such as allergic reactions, liver and kidney toxcity [2]. In recent years, due to its mild drug and overall regulation, traditional Chinese medicine (TCM) has played an increasingly important role in reducing UA and lowering blood lipids [12]. The traditional Chinese prescription Si Miao Wan originated in an ancient Chinese book named “Cheng Fang Bian Du,” consisting of four separate herbs, *Atractylodes*, *Cork*, *Coix* seed, and *Achyranthes*. For centuries, the Chinese have been using it to treat gout and have achieved good results. However, the prescription clinically only uses the adjuvant treatment of acute gouty arthritis due to its drug composition. According to the results of the symptom differentiation, it will be adjusted during the prescription. The original prescription will adjust some drugs [13] to achieve a better therapeutic effect. Due to Si Miao Wan not meeting actual clinical needs, many people have modified the Si Miao Wan based on the theory of TCM [14, 15].

*Dendrobii officinalis* (DOF) Caulis are the dried stems of the orchid *Dendrobium officinalis* Kimura et Migo of the family Orchidaceae. DOF is a traditional Chinese edible plant that has been medicinally used in China for hundreds of years, with a wide range of functions such as protecting against cancer and lowering blood pressure [16]. Prelaboratory studies have found that DOF Chinese herb compound can reduce SUA [13], and it has also been found to effect of regulating blood lipids [17]. Based on Si Miao Wan, we added TCM DOF to form the *Dendrobium officinalis* Six nostrum (DOS). The role and mechanism of DOS in reducing UA and lowering blood lipid not fully understood.

UA production and metabolism are a complex physiological process involving multiple organs such as the liver, kidney, and intestine of the body, enzymes within the organs, kidney and intestinal excretions [18]. HUA is caused due to various factors leading to increased UA production or reduced excretion in the body. Endogenous UA is derived from the metabolism of nucleic acids and other terpenoids in the body, accounting for 80∼90% of the total UA in the body. Also, HUA can be caused by genetic factors which leads to the dysfunction of enzymes related to sputum metabolism. Exogenous UA comes from ingested food, including excessive intake of fructose, fatty foods and alcohol consumption are closely related to HUA [19]. Approximately 2/3 of the excretion of UA is excreted by the kidney, and the remainder is excreted by the digestive tract [20].

Through the above theoretical analysis, we replicated chronic HUA with HPA rat model by using potassium oxide (PO) (200 mg/kg) combined with high-fat sorghum feed. We studied the pharmacological effects and mechanisms of DOS by combining renal and intestinal UA transporters, inflammatory proteins, liver UA synthesis proteins and lipid regulatory proteins.

## 2. Materials and Methods

### 2.1. Materials and Chemicals

High-fat sorghum feed: cooked lard 10%, egg yolk powder 5%, cholesterol 1%, cholate 0.25%, yeast extract 10%, and adenine 0.15% mixed into common feed and repressed into pellets (Jiangsu Nantong Trophy, China). The biochemical reagents total cholesterol (TC), triglycerides (TG), UA, LDL-c, and high-density lipoprotein cholesterol (HDL-c) were purchased from Ningbo Medical System Biotechnology Co., Ltd (Ningbo, China).

Potassium oxonate (PO) was purchased from Shanghai Yuanye Biotech Industry Co., Ltd. (Shanghai, China). Pentobarbital sodium was purchased from Chengdu Huaxia Chemical reagent Co., Ltd. (Chengdu, China). Antibodies, including glyceraldehyde-3-phosphate dehydrogenase (GAPDH), PDZ domain protein kidney 1 (PDZK1), ATP-binding cassette subfamily G member 2 (ABCG2), NACHT-LRR and PYD domains-containing protein 3 (NLRP3), Toll-like receptor 4 (TLR4), Caspase-1, and Hypoxanthine-guanine phosphoribosyl transferase 1 (HPRT1) were purchased from Protein Technology Inc. (MA, USA). Enzyme-linked immunosorbent assay (ELISA) kits with xanthine oxidase (XOD) and adenosine deaminase (ADA) were purchased from Shanghai Yuanye Biotech Industry Co., Ltd. (Shanghai, China). ELISA kits with fatty acid-binding protein (FABP1) and lipoprotein lipase (LPL) were purchased from Shanghai Enzyme Biotechnology Co., Ltd. (Shanghai, China).

### 2.2. Preparation of DOS Extract

Based on the experience of our laboratory in drug extraction process, the medicinal materials in DOS are weighed in a certain proportion and soaked in water overnight, then extracted twice with purified water (sample: solvent, 1 : 10, w/v) under reflux for 2 hours each time. The extract was concentrated at 65°C by rotary evaporator. The extract of high concentration DOS (crude drug concentration 1.38 g/mL) was obtained from the brown colloidal substance. DOS of medium and low concentration was diluted with purified water, respectively.

### 2.3. Animals and Experimental Design

50 specific pathogen-free (SPF) SD rats, with a body mass of 200 ± 20 g, were provided by Zhejiang Animal Experimental Center (license number: SCXK (Zhejiang, China) 2014-0001 and animal quality certificate number: 1809120001). Feeding conditions: indoor temperature 25 ± 1°C, humidity 45 ± 5%, and standard 12 h light/12 h dark rhythm. Rats were allowed free access to food and tap water. All procedures were performed according to protocols following the guidelines for the Use and Care of Laboratory Animals published by the Zhejiang province (2009).

After 1 week of acclimation to the laboratory, 50 male SD rats were randomly divided into five groups: normal control (NC) group, model control (MC) group, high dose of DOS (DOS-H, 13.80 g/kg body weight) group, medium dose of DOS (DOS-M, 6.90 g/kg body weight) group, and low dose of DOS (DOS-L, 3.45 g/kg body weight) group, and each group has 10 rats. We also set up three reference drug groups (benzbromarone group, allopurinol group, and ezetimibe group), but in this article, in order to highlight the effect of DOS, the results of the reference drugs group are not shown. The rat model of HUA with HPA was established by feeding high-fat sorghum feed combined with gavage PO. Four weeks after the establishment of the model, the orbital blood was collected to determine whether the model was successfully prepared. After the model was successfully prepared, the DOS was administered continuously on a daily basis for 8 weeks. At the end of experiment, rats were anesthetized (intraperitoneal injection pentobarbital sodium, 90 mg/kg), the kidney, intestine, and liver tissues were separated as soon as possible, and pictures of the liver were taken. Weigh the liver and calculate the liver index (liver weight/body weight). A part of tissues was put in 10% formalin solution for pathology, and other parts were stored at −80°C for enzyme-linked immunosorbent assay (ELISA) and western blot analysis.

### 2.4. Measuring Blood Lipids and SUA Changes

Serum biochemical indicators were tested every 4 weeks after the start of the experiment. Rats were fasted for 12 hours, then blood was drawn from the ophthalmic venous plexus and centrifuged at 3,000 r/min for 15 min, and serum was separated. The levels of UA, TC, TG, LDL-c and HDL-c in serum were measured by automatic biochemical analyzer (Hitachi 7020, Japan) according to the scheme of the manufacturer of blood biochemical kit.

### 2.5. Histopathological Examination of the Intestine and Kidney

The histopathological examination of the intestine and kidney was evaluated by hematoxylin and eosin (H&E) staining as previously described [16]. The intestinal and kidney tissues from SD rats were immobilized in 10% formalin solution for 48 hours. Fixed samples were processed and paraffin sections with a thickness of 4 *μ*m were prepared. The slides were stained with H&E staining and the histopathological changes were examined by optical microscopy (OLYMPUS BX43, Germany).

### 2.6. Enzyme-Linked Immunosorbent Assay (ELISA)

The liver tissues were weighed, and a volume of saline solution was added and mixed at a ratio (w/v) of 1 : 9. Then, the liver samples were homogenized and centrifuged at 12,000 r/min for 10 min at 4°C. No liver tissue fragments were found in the turbid liquid of the centrifugal tube. The supernatant was centrifuged at 3,500 r/min and 4°C for 15 min and stored for testing [16]. The activities of liver proteins XOD, ADA, FABP1, and LPL were detected by Epoch2 (Bio-TEK, USA). All of the procedures were performed as described in the assay kit.

### 2.7. Immunohistochemistry (IHC) Staining

The intestine and kidney were taken to prepare 4 *μ*m thick paraffin sections. Mouse and rabbit-specific HRP/DAB (ABC) assay IHC kit was used to detect expression of ABCG2, Caspase-1, TLR4, and NLRP3 in kidney and intestine specimens, and the tissue was counterstained with hematoxylin. In brief, kidney sections were incubated with diluted primary antibodies ABCG2 (1 : 200), Caspase-1 (1 : 200), and NLRP3 (1 : 200) overnight at 4°C, and intestinal sections were incubated with diluted primary antibodies ABCG2 (1 : 200), TLR4 (1 : 200), and NLRP3 (1 : 200) overnight at 4°C [16]. Tissue sections were photographed using a biological microscope. The results of IHC staining were expressed as mean integrated optical density and analyzed using Imagine Pro-Plus 6.0 software.

### 2.8. Western Blot Analysis

In brief, the weighed liver and kidney samples were immersed in a 1 mL precooled RIPA (Solarbio, Beijing, China) buffer containing benzenesulfonyl fluoride (PMSF) inhibitor (Beyotime, Shanghai, China) and homogenized at 4°C. The homogenate was centrifuged at a speed of 12,000 r/min for 10 minutes, then the supernatant was collected, and the protein concentration of the sample was detected by BCA protein determination kit (Beyotime, Shanghai, China). Mix the protein sample with the sample buffer in a ratio of 4 : 1 and boil for 10 minutes. The mixture was separated from 10% SDS-PAGE gel and transferred to PVDF membrane (Bio-Rad, USA) for 1 hour. The membrane was sealed in 5% skim milk powder dissolved in TBST for 2 hours [21]. Then, the membrane was incubated overnight with anti-HPRT1 (1 : 1,000), PDZK1 (1 : 1,000), and GAPDH (1 : 8,000) antibodies at 4°C. After washing three times in TBST, the membrane was incubated in a suitable second antibody conjugated with HRP (Beyotime, Shanghai, China) for 2 hours at room temperature and then washed three times in TBST again. Protein bands were displayed by ECL kit (Beyotime, Shanghai, China) and analyzed by Image J software.

### 2.9. Statistical Analysis

In this experiment, SPSS 19.0 software was used for data processing. All data were one-way analysis of variance (ANOVA) expressed by mean ± standard deviation (SD). The LSD *t*-tests were applied when homogeneity of variance assumptions was satisfied; otherwise, the Dunnett *t*-test was used. A value of *P* < 0.05 was considered to be statistically significant. Diagrams were obtained by GraphPad Prism 7.0.

## 3. Results

### 3.1. Effect on SUA Level

**Figure 1:**
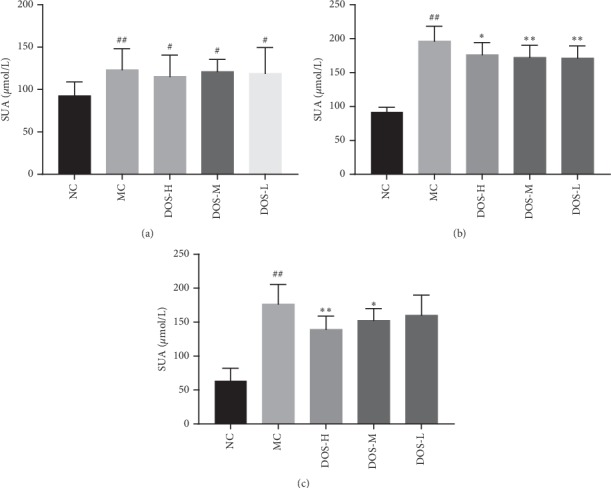
Effect of DOS on SUA. (a) SUA after 0-week administration. (b) SUA after 4-week administration. (c) SUA after 8-week administration. NC—normal control; MC—model control; DOS-L—low dose of DOS (3.45 g/kg body weight); DOS-M—medium dose of DOS (6.90 g/kg body weight); DOS-H—high dose of DOS (13.80 g/kg body weight). The data were expressed as mean ± SD of 8–10 rats in each group. ^#^*P* < 0.05; ^##^*P* < 0.01, compared with normal control group; ^*∗*^*P* < 0.05; ^*∗∗*^*P* < 0.01, compared with model control group.

As shown in Figures 1(a)∼1(c), after modeling for 4 weeks, compared with the NC group, the SUA level of rats in each MC group increased significantly (*P* < 0.01,  *P* < 0.05), suggesting that the SUA level of rats fed with high-fat sorghum feed for 4 weeks could significantly increase. After 4-week administration, compared with the NC group, the SUA level in the MC group increased significantly (*P* < 0.01), with an average increase of 116.3%. Compared with the MC group, the SUA level in the three dosage groups of DOS decreased significantly (*P* < 0.01, *P* < 0.05), with a mean decrease of 10.2∼12.7%. It is suggested that three doses of DOS have significant effect on reducing SUA. After 8-week administration, compared with the NC group, the SUA level in the MC group increased significantly (*P* < 0.01), and the mean value increased by 185.2%. Compared with the MC group, the DOS-H and DOS-M groups could significantly reduce the SUA level in rats (*P* < 0.01, *P* < 0.05), and the mean value decreased by 15.9∼23.2%, but the level of SUA in the DOS-L group was not statistically different.

### 3.2. Effect on Liver Appearance and Serum Lipid Level

**Figure 2:**
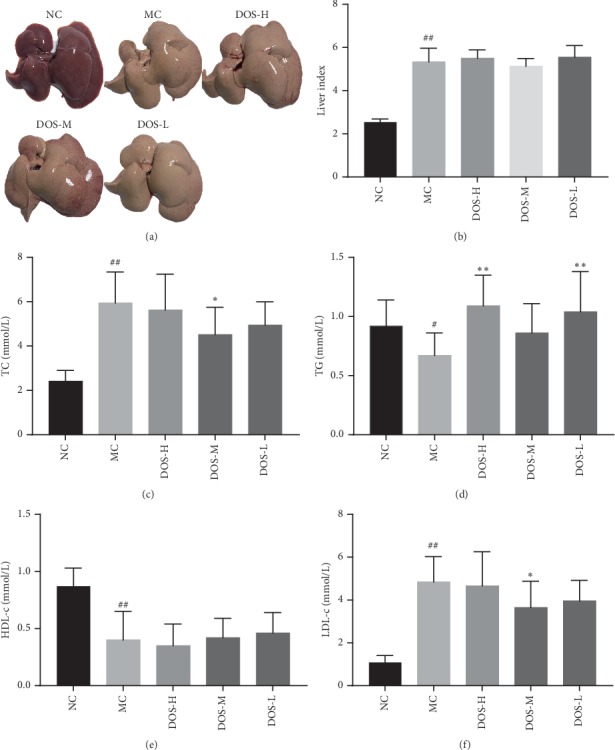
Effect of DOS on blood lipids, liver weight, and appearance. (a) Liver appearance. (b) Liver index. (c) Serum TC. (d) Serum TG. (e) Serum HDL-c. (f) Serum LDL-c. NC—normal control; MC—model control; DOS-L—low dose of DOS (3.45 g/kg body weight); DOS-M—medium dose of DOS (6.90 g/kg body weight); DOS-H—high dose of DOS (13.80 g/kg body weight). The data were expressed as mean ± SD of 8–10 rats in each group. ^#^*P* < 0.05; ^##^*P* < 0.01, compared with normal control group; ^*∗*^*P* < 0.05; ^*∗∗*^*P* < 0.01, compared with model control group.

As shown in Figures 2(a) and 2(b), the liver of the NC group was reddish brown, soft in texture, and the liver weight was equivalent to 2% of body weight. Compared with the NC group, the MC group rats showed white color due to excessive accumulation of fat in the liver cells, and the liver index was significantly increased (*P* < 0.01). After 8 weeks of administration, DOS improved liver steatosis, but there was no significant decrease in liver index.

As shown in Figures 2(c)∼2(f), after 8 weeks of administration, compared with the NC group, the level of serum TC and LDL-c in the MC group was significantly increased (*P* < 0.01), and the level of serum TG and HDL-c was significantly decreased (*P* < 0.01, *P* < 0.05). Compared with the MC group, DOS-M group significantly decreased the level of serum TC and LDL-c in rats (*P* < 0.05), and the mean decreased by 23.9% and 24.7%, respectively; the DOS-H group significantly increased serum TG level in rats (*P* < 0.01), and the mean value is close to the NC group. The DOS-L group can significantly increase serum TG levels in rats (*P* < 0.01).

### 3.3. Histopathological Analysis

**Figure 3:**
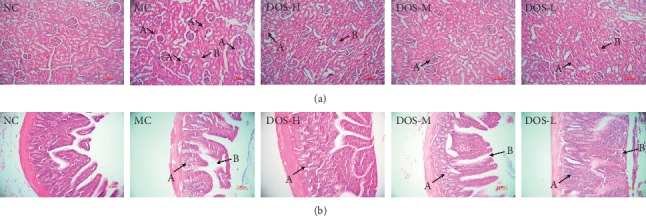
Effect of DOS on kidney and intestinal histopathology of rats (×200). (a) Kidney tissue pictures: A—glomerular atrophy; B—renal interstitial inflammation. (b) Intestinal tissue pictures: A—decreased number of goblet cells in the intestinal; B—intestinal epithelial cells. NC—normal control; MC—model control; DOS-L—low dose of DOS (3.45 g/kg body weight); DOS-M—medium dose of DOS (6.90 g/kg body weight); DOS-H—high dose of DOS (13.80 g/kg body weight). *n* = 8–10.

As shown in Figure 3(a), after 8 weeks of administration, the renal cells in the NC group were structurally intact and neatly arranged, and there was no inflammatory cell infiltration in the renal interstitial. Compared with the NC group, the MC group showed obvious glomerular atrophy (A), and there was obvious inflammatory cell infiltration in the renal interstitial (B). Compared with the MC group, the three doses of DOS significantly reduced the rat kidney damage. It reduces the amount of glomerular atrophy and improves renal interstitial inflammation.

As shown in Figure 3(b), after 8 weeks of administration, the intestinal epithelial cells in the NC group had a clear structure and were neatly arranged. Compared with the NC group, the number of intestinal glandular cells in the MC group decreased (A), and the intestinal epithelial cells partially fell off (B). Compared with the MC group, DOS-H and DOS-M groups both increased the number of glandular cells in the intestinal, and the intestinal epithelial cells in the DOS-H group were structurally intact and neatly arranged.

### 3.4. Effect on the Expression of Inflammatory Protein in Rat Kidney and Intestinal Tissues

After pathological observation of the kidney and intestine, we further studied the level of inflammatory bodies in the kidney and intestinal tissues of rats.

#### 3.4.1. Effect on NLRP3 and Caspase-1 Protein Level in Rat Kidney

**Figure 4:**
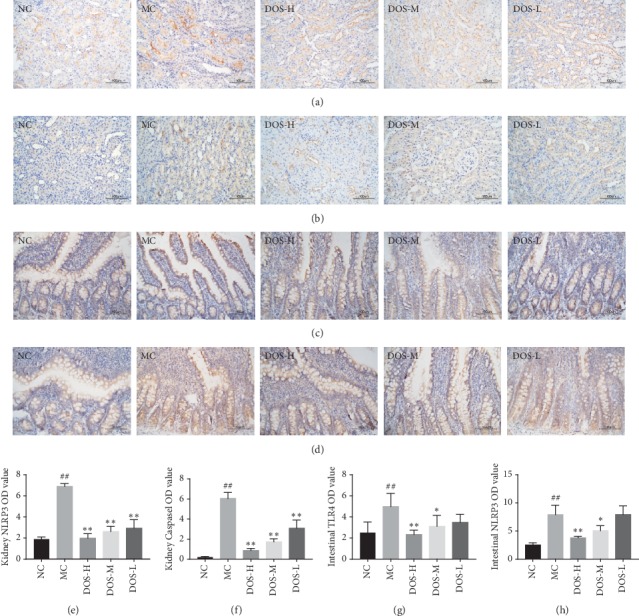
Effect of DOS on inflammatory proteins in kidney and intestinal tissues. (a) Kidney NLRP3 IHC pictures (×400). (b) Kidney Caspase IHC pictures (×400). (c) Intestinal TLR4 IHC pictures (×400). (d) Intestinal NLRP3 IHC pictures (×400). (e) Kidney NLRP3 OD value. (f) Kidney Caspase-1 OD value. (g) Intestinal TLR4 OD value. (h) Intestinal NLRP3 OD value. NC—normal control; MC—model control; DOS-L—low dose of DOS (3.45 g/kg body weight); DOS-M—medium dose of DOS (6.90 g/kg body weight); DOS-H – high dose of DOS (13.80 g/kg body weight). The data were expressed as mean ± SD of 8–10 rats in each group. ^#^*P* < 0.05; ^##^*P* < 0.01, compared with normal control group; ^*∗*^*P* < 0.05; ^*∗∗*^*P* < 0.01, compared with model control group.

As shown in Figures 4(a) and 4(e), after 8 weeks of administration, compared with the NC group, the NLRP3 level in the MC group was significantly increased (*P* < 0.01). Compared with the MC group, the three dose groups of DOS significantly reduced the expression of NLRP3 in the rat kidney (*P* < 0.01).

As shown in Figures 4(b) and 4(f), after 8 weeks of administration, compared with the NC group, the level of Caspase-1 in the MC group was significantly increased (*P* < 0.01). Compared with the MC group, the three doses of DOS significantly decreased the expression level of Caspase-1 in the kidney (*P* < 0.01).

#### 3.4.2. Effect on TLR4 and NLRP3 Protein Level in Rat Intestine

As shown in Figures 4(c) and 4(g), after 8 weeks of administration, compared with the NC group, the level of TLR4 in the MC group was significantly increased (*P* < 0.01). Compared with the MC group, DOS-H and DOS-M groups significantly decreased the expression of TLR4 in the intestine of rats (*P* < 0.01, *P* < 0.05).

As shown in Figures 4(d) and 4(h), after 8 weeks of administration, compared with the NC group, the level of NLRP3 in the intestine of the MC group was significantly increased (*P* < 0.01). Compared with the MC group, DOS-H and DOS-M groups significantly decreased the expression of NLRP3 in the intestine of rats (*P* < 0.01, *P* < 0.05).

### 3.5. Effect on UA Production

**Figure 5:**
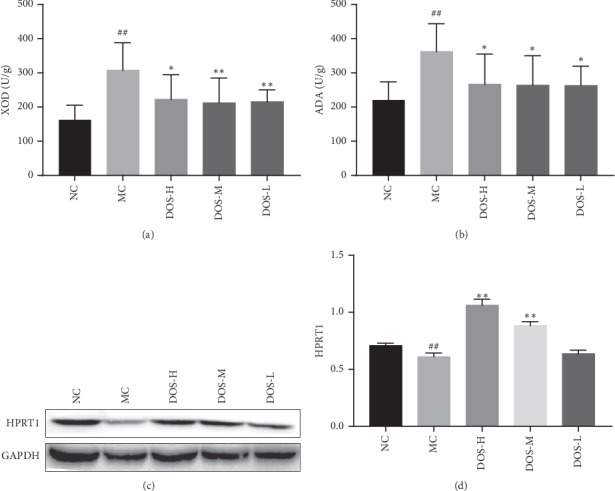
Effect of DOS on UA production. (a) Liver XOD content. (b) Liver ADA content. (c) HPRT1 protein band. (d) HPRT1 protein OD value. NC—normal control; MC—model control; DOS-L—low dose of DOS (3.45 g/kg body weight); DOS-M—medium dose of DOS (6.90 g/kg body weight); DOS-H—high dose of DOS (13.80 g/kg body weight). The data were expressed as mean ± SD of 8–10 rats in each group. ^#^*P* < 0.05; ^##^*P* < 0.01, compared with normal control group; ^*∗*^*P* < 0.05; ^*∗∗*^*P* < 0.01, compared with model control group.

As shown in Figures 5(a) and 5(b), after 8 weeks of administration, compared with the NC group, the level of XOD and ADA in the MC group increased significantly (*P* < 0.01), and the mean values increased by 89.2% and 64.0%, respectively. Compared with the MC group, the three doses of DOS significantly decreased the level of XOD and ADA (*P* < 0.01, *P* < 0.05), and the mean values decreased by 27.7∼30.0% and 26.3∼27.3%.

As shown in Figures 5(c) and 5(d), after 8 weeks of administration, the content of HPRT1 protein in rat liver tissue was analyzed by western blot. The HPRT1 protein expression was significantly downregulated in the MC group compared to the NC group (*P* < 0.01). The DOS-H and DOS-M dose groups effectively upregulated HPRT1 protein expression in the liver (*P* < 0.01), suggesting that DOS may inhibit UA production by increasing HPRT1 activity in the liver.

### 3.6. Effect on UA Excretion

**Figure 6:**
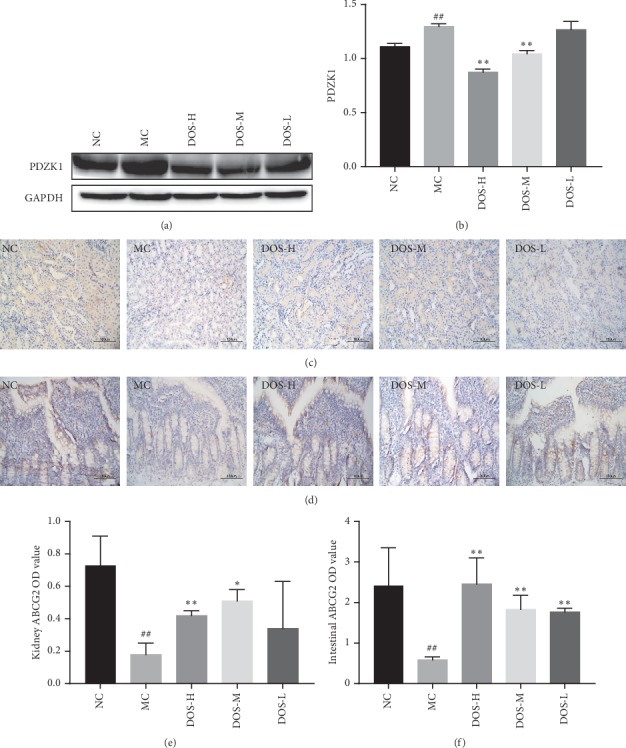
Effect of DOS on UA excretion. (a) PDZK1 protein band. (b) PDZK1 protein OD value. (c) Kidney ABCG2 IHC pictures (×400). (d) Intestinal ABCG2 IHC pictures (×400). (e) Kidney ABCG2 OD value. (f) Intestinal ABCG2 OD value. NC—normal control; MC—model control; DOS-L—low dose of DOS (3.45 g/kg body weight); DOS-M—medium dose of DOS (6.90 g/kg body weight); DOS-H—high dose of DOS (13.80 g/kg body weight). The data were expressed as mean ± SD of 8–10 rats in each group. ^#^*P* < 0.05; ^##^*P* < 0.01, compared with normal control group; ^*∗*^*P* < 0.05; ^*∗∗*^*P* < 0.01, compared with model control group.

As shown in Figures 6(a) and 6(b), after 8 weeks of administration, the content of PDZK1 protein in rat kidney tissue was analyzed by western blot. The PDZK1 protein expression was significantly upregulated in the MC group compared to the NC group (*P* < 0.01). Compared with the MC group, DOS-H and DOS-M groups effectively reduced the expression of PDZK1 in kidney tissue (*P* < 0.01). These results indicate that DOS plays an important role in UA excretion.

As shown in Figures 6(c) and 6(e), after 8 weeks of administration, the content of ABCG2 protein in rat kidney tissue was analyzed by IHC. Compared with the NC group, the level of ABCG2 protein in the MC group was significantly lower (*P* < 0.01). Compared with the MC group, DOS-H and DOS-M dose groups significantly increased the level of ABCG2 protein in the kidney (*P* < 0.01, *P* < 0.05).

As shown in Figures 6(d) and 6(f), after 8 weeks of administration, the content of ABCG2 protein in rat intestinal tissue was analyzed by IHC. Compared with the NC group, the level of ABCG2 protein in the MC group was significantly lower (*P* < 0.01). Compared with the MC group, three dose groups of DOS can significantly increase the level of ABCG2 protein in the intestine (*P* < 0.01).

### 3.7. Effect on Lipid Metabolism

**Figure 7:**
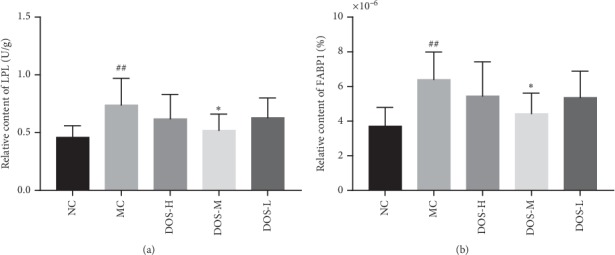
Expression of LPL and FABP1 in rat liver tissue. (a) Liver LPL relative content. (b) Liver FABP1 relative content. NC—normal control; MC—model control; DOS-L—low dose of DOS (3.45 g/kg body weight); DOS-M—medium dose of DOS (6.90 g/kg body weight); DOS-H—high dose of DOS (13.80 g/kg body weight). The data were expressed as mean ± SD of 8–10 rats in each group. ^#^*P* < 0.05; ^##^*P* < 0.01, compared with normal control group; ^*∗*^*P* < 0.05; ^*∗∗*^*P* < 0.01, compared with model control group.

After 8 weeks of administration, the content of LPL and FABP1 protein in rat liver tissue was analyzed by ELISA kit. As shown in Figures 7(a) and 7(b), compared with the NC group, the LPL and FABP1 levels in the liver tissue of the MC group were significantly increased (*P* < 0.01). Compared with the MC group, the LPL and FABP1 levels in the liver tissue of the DOS-M group were significantly lower (*P* < 0.05), suggesting that the DOS-M group may regulate blood lipid level by reducing the level of LPL and FABP1 in the liver tissue. This result also explains that only DOS-M can reduce rat serum TC and LDL-c.

## 4. Discussion

Long-term intake of foods with excessive strontium content such as seafood, beer, and animal offal will abnormally increase the activity of liver UA synthase, thereby metabolizing excessive UA [22]. Long-term consumption of foods high in fat content will lead to elevated blood lipid level and induce liver steatosis [23, 24]. Considering the presence of uricase in rodents, the UA enzyme inhibitor PO can be used to eliminate the effects of UA in rats, making the model closer to the pathogenesis of human HUA with HPA [25]. We used high-fat sorghum feed and PO to induce an increase in SUA and blood lipids level in rats. After 4 weeks under experimental conditions, the results showed that SUA was significantly elevated and abnormal lipid metabolism was disordered. On the other hand, this model may cause the liver to become heavier and the color to whiten. Therefore, we conclude that PO combined with high-fat sorghum feed can produce a suitable model that mimics the pathogenesis of humans and can be used to study the causes of metabolic disorders involving HUA with HPA. This model can be used to study pathogenesis and screen for potential therapeutic targets. The main indicator for the diagnosis of HUA is the abnormal increase in SUA levels. The reason for this phenomenon is due to excessive UA production or reduced UA excretion [1]. TC, TG, HDL-c and LDL-c are important biochemical indicators for detecting HPA [26]. This study shows that DOS can reduce the level of UA, TC and LDL-c in serum and can improve the fatty degeneration of the liver to a certain extent.

It has been reported that UA can induce activation of inflammatory bodies. NLRP3, Caspase-1 and TLR4 are critical inflammatory mediators involved in the body's inflammatory response. When the inflammatory bodies are activated, it will cause a large amount of release of various other inflammatory factors, aggravating tissue cell damage [27, 28]. Long et al. [29, 30] found that HUA triggers renal inflammation in rats by activating NLPR3 inflammasome in the rat kidney. Additional studies have shown that UA regulates the expression of NLRP3 inflammasome in intestinal epithelial cells by activating the TLR4 pathway, causing intestinal damage [27]. The experimental results showed that continuous DOS administration for 8 weeks effectively inhibited kidney inflammation, inhibited renal lesions, and maintained normal renal UA transport and excretion by inhibiting renal NLRP3 inflammasome expression. At the same time, continuous administration of DOS for 8 weeks may reduce the expression of NLRP3/Caspase-1 inflammasome in intestinal cells by inhibiting the expression of inflammatory pathway of intestinal TLR4 and relieving intestinal inflammatory injury and enhancing intestinal UA excretion.

UA is end product of the body's metabolism, and the liver is the primary place for UA production. XOD and ADA are two key enzymes in the UA pathway that are metabolized by the liver [31, 32]. Increased activity of XOD and ADA promotes catabolism of nucleic acids and increases UA production. The results of this experiment showed that continuous administration of DOS for 8 weeks significantly reduced the activity of XOD and ADA in the liver of model rats. Continuous administration of DOS for 8 weeks may reduce liver UA production and lower SUA level by inhibiting liver sputum metabolic enzyme activity.

HPRT1 is an enzyme that converts guanine to guanosine monophosphate and converts hypoxanthine to inosine monophosphate. It is carried by transferring the 5-phosphate ribose group of 5-phosphoribosyl pyrophosphate to the guanidine and plays a central role in the production of purine nucleotides through the purine rescue pathway. Partial defects in HPRT1 enzymatic activity can lead to clinical symptoms such as HUA, urate kidney stones, and gout [33, 34]. The results of this experiment showed that continuous administration of DOS for 8 weeks significantly increased the activity of HPRT1 in the liver of model rats. It suggests that DOS may reduce the production of UA in the liver and lower the SUA level by upregulating the activity of HPRT1.

SUA level depends on the balance of UA production and excretion in the body [18]. The kidney is the main organ for UA excretion in the body. 70% of UA in the human body is excreted by the kidney. UA is processed in the kidney through glomerular filtration, renal tubular reabsorption, and resecretion. UA is excreted in the kidney through glomerular filtration, reabsorption of renal tubules (collection tubes), and re-secretion after reabsorption; among them, UA transport proteins play a vital role in uric acid excretion [35]. ABCG2 plays a crucial role in kidney excretion. As an ATP-binding transporter, ABCG2 is distributed in the proximal ductal cells of the kidney and has the function of secreting UA [36, 37]. The results of this experiment show that DOS-H can significantly increase the activity of ABCG2 in rat kidney.

The intestine is the most important UA excretion organ, after the kidney. The main UA excretion organ in patients with clinical renal failure is the intestine. ABCG2 is an important urate transporter in the intestine [20]. A decrease in ABCG2 protein activity inhibits intestinal UA excretion and causes HUA. The results of the experiment showed that continuous DOS administration for 8 weeks significantly increased the level of ABCG2 in the intestine of rats. It can be seen that DOS improves the UA intestinal excretion ability and lowers the rat SUA level by enhancing intestinal ABCG2 activity.

PDZK1 is encoded by the PDZ-containing domain 1 gene and is a scaffold protein of various transporters and regulatory proteins. PDZK1 is expressed in the kidney, liver, intestine and adrenal cortex [38]. PDZK1 has the function of regulating several transport proteins, including UA transporters ABCG2 [38–40]. Yang et al. [41, 42] found that HUA can upregulate the expression of PDZK1 in rat kidney. The analyses from this part of the experiment showed that continuous DOS administration for 8 weeks significantly reduced the level of PDZK1 in rat kidney. In short, DOS can increase the content of ABCG2 in the kidney and intestine, reduce the content of PDZK1 in the kidney, and result in the reduction the level of serum UA.

We found that DOS has the role of regulating lipid metabolism, and we explored the relevant mechanisms. LPL is a key enzyme regulating lipoprotein metabolism. Under the action of LPL, there is an increase in the degradation of TG in the blood, the conversion or metabolism of TC, increase in the HDL in the blood, and the release of free fatty acids by hydrolysis [43]. However, LPL can promote the uptake and storage of free fatty acids by fat cells, which is one of the critical factors leading to obesity and associated diseases [44]. In this experiment, the rat liver tissue contained a large amount of fat, so LPL may be overexpressed in adipocytes. The experimental results also confirmed that the LPL content of the model rats was higher than expected. The DOS improved liver tissue steatosis in the model rats, which in turn reduced the LPL content in the liver tissue. FABP1 is the most expressed FABP molecule in human body and is mainly distributed in the liver, kidney, lung and gastrointestinal tract [45]. FABP1 transports plasma fatty acids into cells and converts them into triglycerides and phospholipids. Studies have shown that a high-fat diet increases liver FABP1 content, leading to high expression of FABP1 gene. FABP1 inhibits VLDL production, affecting endogenous TG transport and accumulation of TC in serum. Our experimental results show that DOS can inhibit the overexpression of FABP1 gene in model rats, regulate the metabolism of fatty acids in cells, and maintain the relative balance of fatty acid metabolism in the body.

## 5. Conclusion

In conclusion, the results indicate that DOS may promote UA excretion, inhibit UA production, and promote lipids metabolism. In addition, DOS can also improve the pathological morphology of the kidney and intestine by reducing the activation of inflammatory bodies and achieving the goal of treating HUA with HPA. This article suggests the advantages of traditional Chinese medicine. This article also provides a basis for the treatment of HUA with HPA using traditional Chinese medicine.

## Data Availability

The data used to support the findings of this study are available from the corresponding author upon request.
